# Rigorous assessment and integration of the sequence and structure based features to predict hot spots

**DOI:** 10.1186/1471-2105-12-311

**Published:** 2011-07-29

**Authors:** Ruoying Chen, Wenjing Chen, Sixiao Yang, Di Wu, Yong Wang, Yingjie Tian, Yong Shi

**Affiliations:** 1 College of Life Sciences, Graduate University of Chinese Academy of Sciences, Beijing 100049, China; 2 Research Center on Fictitious Economy and Data Science, Chinese Academy of Sciences, Beijing 100190, China; 3 Department of Biomedical Engineering, College Life Science and Technology, Tongji University, Shanghai 200092, China; 4 Academy of Mathematics and Systems Science, Chinese Academy of Sciences, Beijing 100190, China; 5 College of Information Science and Technology, University of Nebraska at Omaha, Omaha NE 68182, USA

## Abstract

**Background:**

Systematic mutagenesis studies have shown that only a few interface residues termed hot spots contribute significantly to the binding free energy of protein-protein interactions. Therefore, hot spots prediction becomes increasingly important for well understanding the essence of proteins interactions and helping narrow down the search space for drug design. Currently many computational methods have been developed by proposing different features. However comparative assessment of these features and furthermore effective and accurate methods are still in pressing need.

**Results:**

In this study, we first comprehensively collect the features to discriminate hot spots and non-hot spots and analyze their distributions. We find that hot spots have lower relASA and larger relative change in ASA, suggesting hot spots tend to be protected from bulk solvent. In addition, hot spots have more contacts including hydrogen bonds, salt bridges, and atomic contacts, which favor complexes formation. Interestingly, we find that conservation score and sequence entropy are not significantly different between hot spots and non-hot spots in Ab+ dataset (all complexes). While in Ab- dataset (antigen-antibody complexes are excluded), there are significant differences in two features between hot pots and non-hot spots. Secondly, we explore the predictive ability for each feature and the combinations of features by support vector machines (SVMs). The results indicate that sequence-based feature outperforms other combinations of features with reasonable accuracy, with a precision of 0.69, a recall of 0.68, an F1 score of 0.68, and an AUC of 0.68 on independent test set. Compared with other machine learning methods and two energy-based approaches, our approach achieves the best performance. Moreover, we demonstrate the applicability of our method to predict hot spots of two protein complexes.

**Conclusion:**

Experimental results show that support vector machine classifiers are quite effective in predicting hot spots based on sequence features. Hot spots cannot be fully predicted through simple analysis based on physicochemical characteristics, but there is reason to believe that integration of features and machine learning methods can remarkably improve the predictive performance for hot spots.

## Background

A lot of biological processes are regulated or performed by protein-protein interactions [1-5]. Elucidating the molecular mechanism of proteins interactions is a key topic in protein function study. Hence, to fully understand or control biological processes, we need to probe the principles of protein-protein interactions. However, the affinity and specificity in protein-protein interfaces are still poorly understood and many fundamental problems are yet to be solved. In the past years, much efforts have been directed towards the characteristics of protein-protein interfaces, such as electronics interaction, van der Waals forces, shape complementary, residue frequencies, residue-residue contact preferences, and so on [6-12]. Our understanding of protein-protein interfaces benefits greatly from structural analysis, biophysical, or biochemical properties of protein-protein interfaces. Importantly, it has been pointed out that only a few interface residues are central to the binding energy of protein-protein complexes [13,14]. Identifying some key residues that are responsible for protein association can provide important clues for drug design or the causes of many diseases, and a stepping-stone for important applications such as interface redesign.

Large-scale mutation studies have indicated that, "hot spots"- the subset of interface residues, bear most of energetic cost of binding [13-15]. Alanine scanning mutagenesis is the most widely used technique for identifying hot spot residues. When these hot spot residues have been mutated to alanine, they would lead to a striking loss in binding free energy [14]. The role of residues surrounding hot spots has not been well understood until now, and these residues perhaps create a suitable environment for the binding of subunits [14,16,17]. Many studies have demonstrated that most interface residues could be mutated without changing the affinity of proteins complexes [13,18]. Systematic analyses have shown that hot spot residues are abundant in Tyr, Trp, and Arg [14,19]. Lise et al [20] have analysed the distribution of amino acids in hot spots, and found that Trp, Tyr, and Lys appear more frequently in hot spots, which is similar to Bogan's conclusion [14]. It has been shown that hot spots are not evenly distributed along the protein interfaces; rather they are clustered within locally tightly packed regions in the core of the interface. Within the dense clusters, they form a complicated network of interactions and consequently contribute to the stability of the complex; however the contributions of independent clusters are additive [21].

Our major focus in this study is to computationally predict these hot spots in protein-protein interfaces. The prediction of hot spot residues is a difficult but significant problem. As we all know, alanine scanning experiments are time consuming, labor-intensive, and unfeasible on a large scale. Fortunately, computational and theoretical approaches can predict protein-protein interactions sites based on sequence or structure data [22-38]. They can provide valuable information that are complementary to experiments, and give insight into the nature of macromolecular complexes association. Currently, these prediction methods are mainly based on the differences between the characteristics of interface and non-interface residues. However, these approaches cannot predict what residues contribute significantly to the binding free energy. The reason is that, there are no general patterns of physicochemical features, such as evolutionary conservation score, accessible surface area, or secondary structure that can be used for predicting hot spots [15,19,39,40]. Although hot spots cannot be well explained through simple analysis based on the physicochemical characteristics of protein complexes, we still have reason to believe that more computational and theoretical methods will successfully predict them. Some approaches based on rigorous theoretical analysis, but that are validated against the large body of available experimental data, will eventually provide us with a comprehensive understanding of hot spots. Even in advance of such understanding, new experimental techniques will enable development of therapeutics that specifically target protein interfaces hot spots [17].

In recent years, with the growth of experimental data, an increasing number of computational approaches have been developed to predict hot spots in protein-protein interfaces. One class is based on the energy such as computational alanine scanning approach, which uses free energy functions (including van der Waals potentials, electrostatic interactions, hydrogen bonds, and desolvation energy) to calculate the change of binding free energy [41-47]. A second class combines various features of residues with machine learning approaches. Darnell et al [48,49] used decision tree approach to predict protein-protein interaction hot spots based on the shape specificity and biochemical contact. Cho et al [50] performed feature selection from 54 multifaceted features using decision tree, and then modeled protein-protein interaction hot spots using support vector machine. Lise et al [20] developed a hybrid scheme to identify hot spots. They considered the basic energy terms as input features of machine learning models such as Support Vector Machines and Gaussian Processes. This approach combines the strengths of machine learning and energy-based methods. In other approaches, Li et al [51] identified hot spot residues at protein-protein interface by examining inter-sidechain interactions. Grosdidier et al [52] predicted hot spots using Normalized Interface Propensity (NIP) values derived from rigid-body docking simulations with electrostatics and desolvation scoring. Finally, hot spots predictions from evolutionary information such as sequence profile and evolutionary conservation score have also been reported [29,39,53,54]. The structural and physicochemical features are informative, and it has been pointed out that each feature cannot solely define hot spots.

Here, we develop a new method, sequence-based support vector machines (SVMs), to identify hot spots in protein-protein interfaces. Different features are combined to improve the hot spots prediction performance. These various features are extracted from protein sequences and structures. It is found that the combination of sequence-based features surpasses other combinations in prediction performance. The structure-based method has also relatively high predictive accuracy. We compare our proposed method with other machine learning models and two energy-based approaches. The results demonstrate that our approach is remarkably accurate than other approaches for identifying hot spots. Specifically, our method achieves a precision of 0.69, a recall of 0.68, an F1 score of 0.68, and an AUC of 0.68 on independent test set, respectively. In two case studies, our approach outperforms two energy-based approaches with high accuracy.

Additionally, we also analyze the distributions of some features between hot spots and non-hot spots. Our results show that lower relASA and larger relative change in ASA are critical for hot spots distinguishing from non-hot spots. In Ab+ dataset (all complexes), the statistically differences in conservation score and sequence entropy between hot spots and non-hot spots are not significant. However in Ab- dataset (excluding antigen-antibody complexes), there are significant differences. Interestingly, single conservation score or sequence entropy is not a good feature discriminating hot spots from non-hot spots. The performance is remarkably improved when both of them are combined with other features.

## Methods

### Training set

The data set includes 25 protein complexes whose three-dimensional structures are available from Protein Data Bank [55]. Alanine mutation data are collected from the Alanine Scanning Energetics database (ASEdb) [18], the Binding Interface databases (BID) [56] and previous publications [57-62]. To ensure that our data set is sufficiently diverse, we calculate the sequence identity using the PISCES sequence culling server [63]. The sequence identity of at least one protein involved less than 35% is required as in the procedure of previous studies [48,64]. The resulting data set consists of 377 mutated interface residues from 25 protein complexes. The 25 protein structures with resolution ≤3 Å in our data set are listed in Additional file 1: Table S1. The ΔΔ G values (the difference in binding energy between wild-type and mutated protein complex) are also reported in Additional file 1: Table S1. The interface residues are defined as those having Δ*ASA *≥1 Å^2 ^as the definition adopted by Cho [50]. When a residue with ΔΔ G ≥ 1 kcal.mol^-1 ^is defined as a hot spot residue, 377 interface residues contain 182 hot spots and 195 non-hot spots.

### An independent test set

In order to validate our model, an independent test set is collected from the BID. The test set is selected for identical sequence in a similar manner to the training set. Each protein structure has experimentally mutated data but not with ΔΔ G values. In the BID, the effect of a mutation is classified as Strong, Intermediate, Weak, Insignificant, Negative-weak, or Negative-strong. When both strong and intermediate mutations are considered as hot spots, the test data contains 23 complexes including 148 alanine-mutated residues, of which 80 residues are hot spots and 68 residues are non-hot spots. The list of test set is available in Additional file 2: Table S2.

### Collection of features

#### Relative change in ASA and relASA

The solvent accessible surface area (ASA) of each residue is calculated using the program NACCESS [65] with a probe ball radius of 1.4 Å. The ΔASA is the ASA change of a residue upon protein complex formation from monomer state, ΔASA = ASA_monomer _- ASA_complex_, the ASA of a residue in the monomer and complex form, respectively. A residue with ΔASA≥1 Å^2 ^is defined as an interface residue. The relative change in ASA for a residue is calculated as follows: ΔASA%= (ASA_monomer _- ASA_complex _)/ASA_monomer _× 100%. A previous study also referred to this relative surface area burial [50]. Those absolute change in ASA (ΔASA) and solvent accessibility may distinguish hot spots from non-hot spots with a limited capacity. Instead, we use this relative surface burial, simply expressed as ΔASA%. The relative ASA (relASA) of each residue in complex is calculated as the accessibility compared to the accessibility of that residue type in an extended ALA-x-ALA tripeptide (for amino acids) [66].

#### Biochemical contacts

The WHAT IF Servers [67] is used to assess non-covalent interactions in protein complexes. Three types of non-covalent interactions are recorded: hydrogen bonds, salt bridges, and atomic contacts. A hydrogen bond is identified by an optimizing hydrogen-bond networks model [68]. The number of hydrogen bonds that a residue makes with its binding partner is regarded as the residue's hydrogen bond feature. If the distance between a negative atom and a positive atom, one from each side, is less than 7 Å, a salt bridge is evaluated. The number of salt bridges that a residue contacts with its binding partner is considered as the residue's salt bridges feature. If the distance between two atoms, one from each side, is less than 0.25 Å, an atomic contact is identified. Similarly, the number of atomic contacts between a residue and its binding partner is regarded as the residue's atomic contacts feature. The biochemical contacts feature has been used in [48] to predict hot spots.

#### Physicochemical characteristics

The six physicochemical characteristics of an amino acid are hydrophobicity, hydrophilicity, polarity, polarizability, propensities, and average accessible surface area. Deng et al have predicted the protein interaction sites by the physicochemical features and other features [32]. The values of six physicochemical characteristics for each residue are obtained from the AAindex database [69,70].

#### Evolutionary conservation score

Evolutionary conservation score is based on the phylogenetic relations between its close sequence homologues. More conserved positions have higher scores. In this study, we use the color scale to represent the conservation score (e.g. 9-conserved, 1-variable). The evolutionary conservation profiles are obtained from ConSurf Server Database [71]. Similar amino acid sequences in PDB [55] are collected by using PSI-BLAST [72,73] and aligned using MUSCLE [74,75]. The evolutionary conservation of each amino acid position in the alignment is calculated by the Rate4Site algorithm [76,77]. This algorithm takes into account the phylogenetic relations between the aligned proteins and the stochastic nature of the evolutionary process.

#### Sequence entropy

Sequence entropy value for each residue is obtained from HSSP database [78-81]. The sequence entropy shows the conservation at each residue position in a multiple alignment. Every value is normalized over the range 0-100, and the lower sequence entropy values are, the more conserved positions are [82].

#### Sequence profile

Sequence profile is obtained by PSI-BLAST [72] searching against NCBI non-redundant database. The BLOSUM62 substitution matrix and E-value threshold of 0.001 are chosen as parameters. In other words, sequence profile is a Position-Specific Scoring Matrix (PSSM), which is a type of scoring matrix and taken from multiple sequence alignment. In this matrix, amino acid substitution scores are given separately for each position in a protein multiple sequence alignment. PSSM scores are generally shown as positive or negative integers. Positive scores indicate that the given amino acid substitution occurs more frequently in the alignment than expected by chance, while negative scores indicate that the substitution occurs less frequently than expected. The profile value is normalized in the range 0-1 according to the proposed method by Kim et al [83]:(1)

Where*x *is the original value from position specific scoring matrix.

### Support vector machine models

Support Vector Machines (SVMs) [84] are a class of supervised learning algorithms, and can learn a linear decision boundary to discriminate different classes with maximum margin. In bioinformatics and computational biology areas, SVMs have received more and more attentions. For example, SVMs have been applied in the prediction of protein interaction sites and hot spots [20,32,34,36,50].

In the standard SVM, the decision function sgn*f*((*wx*) +*b*) is decided by the following optimization problem:(2)(3)(4)

In our experiment, SVM classifiers are constructed using each feature or the combinations of different features. And we find that the best results are obtained with the radial basis function as the kernel and a set of sequence-based features. The SVM classifiers are implemented on Matlab platform. For each classifier, we use a grid search to determine the optimal values of regularization parameters* C *and γ. The predictive performance of our approach is evaluated by self-consistency test and ten-fold cross-validation test on training set. Also, we validate our approach on an independent data set.

### Evaluation of prediction results

Firstly, the predictive performance of the proposed method is evaluated by self-consistency test on training set. Then, 10-fold cross -validation test is used to evaluate the performance of our method. The data set is randomly divided into ten equal subsets. For each time, nine subsets are used as training data and the remaining subset is used as test data. The following measures are used to evaluate the performance: precision, recall, F1 score, and AUC.(5)(6)(7)

In above equations, TP, FN, TN, and FP are true positives, false negatives, true negatives, and false positives, respectively. Precision is the fraction of predicted hot spots that are true hot spots. Recall is the fraction of true positive hot spots that are predicted hot spots. F1 score is a measure to balance recall and precision rates. In addition, we plot receiver operating characteristics (ROC) curve to evaluate performance. A ROC curve is plotted with true positives rate versus false positives rate for different classification thresholds. The normalized area under a ROC curve (AUC) can measure the classifier's performance.

## Results

### Statistics on the relASA

**Figure 1:**
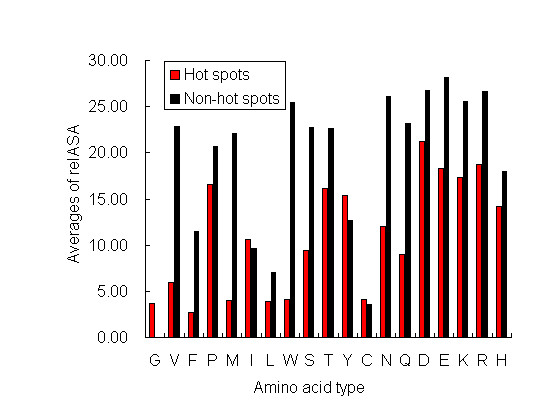
**Averages of relASA for amino acids in hot spots and non-hot spots**. For each type of residue, the average value of its relative ASA in hot spots or non-hot spots is calculated. In 19 amino acids, only G doesn't appear in non-hot spots of our dataset.

The distributions of relASA for each reside in hot spots and non-hot spots are calculated. The average relASA of each amino acid in hot spots and non-hot spots is shown in Figure 1. Because other amino acids are mutated into alanine, alanine doesn't appear in our data set. In 19 common amino acids, only G doesn't appear in non-hot spots of our data set. As shown in Figure 1, except I, Y, and C, the average relASA of each residue in hot spots is lower than that of each residue in non-hot spots. Our results are in good agreement with previous studies, which indicates that hot spots are buried in the complexes (lowASA) [21]. Bogan & Thorn [14], in their influential study hot spot anatomy, noted that hot spots tend to cluster in the center of the interface rather than at the rim, and largely protected from contact with bulk solvent.

### Statistics on relative change in ASA

**Figure 2:**
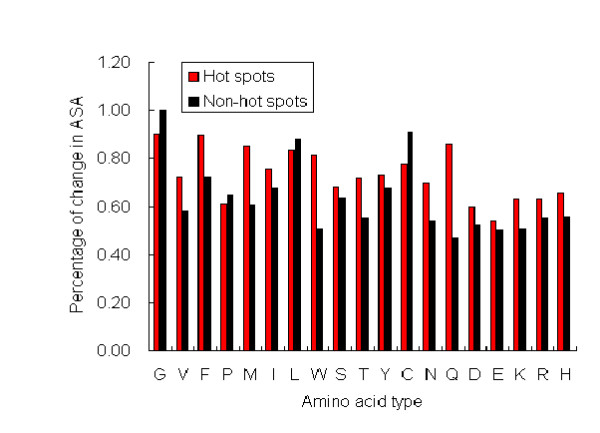
**The relative change of each amino acid in hot spots and non-hot spots**. For each type of residue, its average percentage of change in ASA in hot spots or non-hot spots is shown.

We analyze the relative difference in ASA for each residue type in hot spots and non-hot spots. The results are shown in Figure 2. We find that except G, L and C, the average percentages of change in ASA for hot spots are higher than that of non-hot spots. This suggests that the degree of change in ASA for hot spot residues is stronger. After protein-protein binding, the hot spot residues may disappear on the surface and participate in contacting with residues from partners. Cho el at [50] have chosen this feature to study proteins interaction hot spots, and found that relative surface area burial can distinguish hot spots from non-hot spots.

### Statistics on biochemical contacts

**Figure 3:**
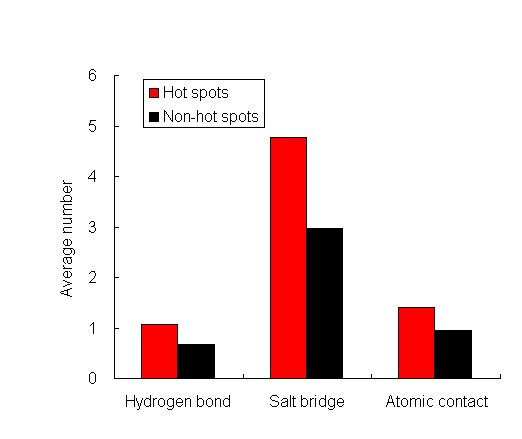
**The biochemical contacts in hot spots and non-hot spots**. The average number of biochemical contacts in hot spots or non-hot spots including hydrogen bonds, salt bridges, and atomic contacts is shown respectively.

We only focus on three kinds of biochemical contacts: hydrogen bonds, salt bridges, and atomic contacts. As can be seen from Figure 3, the average numbers of three classes for hot spots are higher than those of non-hot spots. Hydrogen bonds and salt bridges contribute significantly to the binding free energy. As we all know, electrostatics interactions owe to salt bridges forming. Electrostatics energy and hydrogen energy are important energy terms in free energy calculation.

### Statistics on the distributions of amino acids

**Figure 4:**
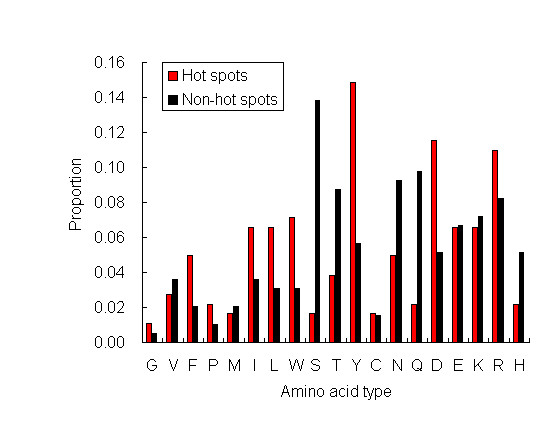
**Distributions of 19 amino acids in hot spots and non-hot spots**. For each type of residue, its percentage among all the residues that are designated as hot spots or non-hot spots is displayed

The distributions of amino acids in hot spots and non-hot spots are shown in Figure 4. Amino acid residues F, P, I, L, and W appear more frequently in hot spots. This indicates that hot spot residues are more likely to be hydrophobic. This is consistent with the O-ring hypothesis that bulk solvent is occluded from hot spots [14]. Amino acids S, T, N, and Q occur more frequently in non-hot spots. This indicates that non-hot spots are more likely to be polar. However, polar residue Y is exceptional, and hot spots are enriched in Y. Amino acid Y can form aromatic π-interactions, and has large hydrophobic surface. In addition, Y is capable of forming one hydrogen bond. Hot spots are also abundant in charged residues D and R. Both of residues can form salt bridges across protein-protein interfaces. As shown in Figure 3, the average number of salt bridges for hot spots is larger than that of salt bridges for non-hot spots.

### Statistics on the evolutionary information

**Figure 5:**
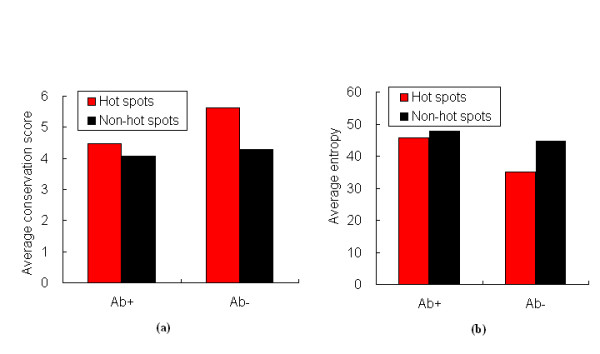
**Comparisons of hot spots and non-hot spots on Ab+ dataset and Ab- dataset**. (a) Average conservation score. (b) Average sequence entropy.

In general, hot spot residues are more conserved than non-hot spot residues. However, our results show that hot spots are not more conserved than non-hot spot residues (Table 1). In Ab+ dataset (all complexes), the averages for hot spots and non-hot spots are 4.46 and 4.08, respectively. The statistically difference in conservation score between hot spots and non-hot spots is insignificant (p -value = 0.14). It is probably that Ab+ dataset includes antigen-antibody complexes, and antibodies must be diversified and easily mutated to recognize externally different antigens. Therefore, evolutionary conservation score may not be a better predictor in distinguishing hot spots from non-hot spots only by itself. Interestingly, when analyzing Ab- dataset (excluding antigen-antibody complexes), we find that the difference in conservation score between hot spots and non-hot spots is statistically significant (p-value = 10^-4^). The mean values for hot spots and non-hot spots are 5.63 and 4.28, respectively. In this case, hot spots are more conserved than non-hot spots. These results are displayed in Figure 5a. But the evolutionary information is insufficient to predict hot spots in protein interfaces. It can combine with other features to improve the predictive performance of models.

**Table 1:** Statistical analysis on evolutionary conservation score and sequence entropy between hot spots and non-hot spots

	Evolutionary conservation score		Sequence entropy	
	
	Ab+	Ab-	Ab+	Ab-
Hot spots^a^	4.46	5.63	45.84	35.14
Non-hot spots^b^	4.08	4.28	47.87	44.59
P-value	0.14	10^-4^	0.36	1.1 × 10^-3^

Ab+ represents the database contains all complexes. Ab- represents the database excludes antigen-antibody complexes.

^a ^Mean value of hot spots.

^b ^Mean value of non-hot spots

### Statistics on the sequence entropy

We also analyze sequence entropy. As can be seen from Figure 5b, not only Ab+ dataset but also Ab- dataset has the similar trend, that is the mean value of sequence entropy for hot spots is lower than that of sequence entropy for non-hot spots. In Ab+ dataset, the p-value of the difference in sequence entropy is 0.36 (Table 1), which indicates that this feature doesn't differ significantly between hot spots and non-hot spots. In Ab- dataset, however p-value for sequence entropy is 1.1 × 10^-3^, implying the difference between hot spots and non-hot spots is statistically significant.

### Training SVM models for different feature combinations

In many studies on predictions of protein interaction sites, different features have been combined to improve the performance of models. These features combinations are as follows: evolutionary profile and accessible surface area (ASA) [85]; physicochemical features, evolutionary conservation score, amino acid distance and position specific scoring matrix (PSSM) [33]; sequence profile and evolutionary rate [23]; PSSM, ASA, and normalized atom contacts [32]; ASA, secondary structure, conservation score, and sequence/spatial distance [36]; temperature factor, sequence profile, and ASA [34]. On the other hand, predictions of hot spots with structure-based or sequence-based methods are also paid more attention. Gao et al analyzed hot spot residues at protein-protein interfaces by using hydrogen bonds, hydrophobic, and van der Waals interaction [86]. Grosdidier and Fernandez-recio applied computational docking to identify hot spots [52]. A good example of sequence-based methods is the one proposed by Ofran and Rost [29]. In their experiment, evolutionary profile, predicted secondary structure, and accessibility to the solvent were combined to predict hot spots. All features were generated from amino acid sequences, suggesting that hot spots have been carved into sequence information without structure features. There are many studies with combinations of structure-based features, sequence-based features, and physicochemical features [48,50,87].

**Figure 6:**
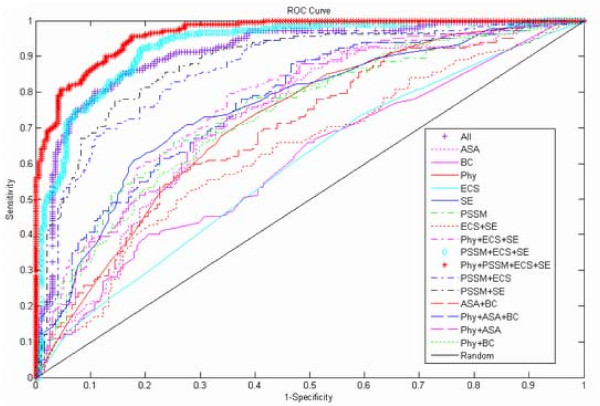
**ROC curves for different models on self-consistency test**. The curves indicate the AUC obtained from different models for different feature combinations with self-consistency test on training set. Each abbreviation is explained simply in Table 2 caption. All indicates that all features are included.

**Figure 7:**
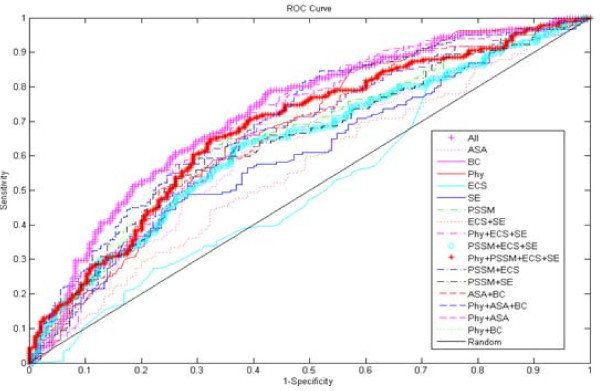
**ROC curves for different models on 10-fold cross-validation test**. The curves for different models are presented on 10-fold cross-validation test.

Following above mentioned publications, we combine different features to train predictive models. Moreover, we compare single feature models to illustrate the discrimination performance of each feature. In total the training set comprises 377 mutations, of which 182 mutations correspond to hot spots. We train different SVM classifiers with different feature combinations. As we know, the structure information sometimes cannot be obtained since many structures of proteins have not been resolved. In PDB, there are 68,840 structures until Oct 26^th^, 2010. On the other hand, the evolutionary information is also unavailable if homologous proteins don't exist. To handle this problem of incomplete information, we construct two models: sequence-based SVM model and structure-based SVM model. The sequence-based model utilizes physicochemical features, PSSM, evolutionary conservation score, and sequence entropy, which comprises no structure information. And the structure-based model uses physicochemical features, ASA, and biochemical contacts without sequence information. The prediction results are compared by precision (P), recall (R), F1-score (F1), and AUC (area under ROC curve). In our work, F1 and AUC bear importance, since F1 score measures the balance precision and recall rates and AUC is independent of any decision threshold. The detailed results of self-consistency test on training set are listed in Table 2, and the ROC curves are displayed in Figure 6. The results of 10-fold cross-validation test are reported in Table 3, and the ROC curves are displayed in Figure 7. On 10-fold cross-validation test, we try many other feature combinations, but only give the better results.

**Table 2:** The results of different models with self-consistency test on training set

Features	P	R	F1	AUC
ASA	0.67	0.70	0.68	0.72
BC	0.64	0.36	0.46	0.61
Phy	0.66	0.63	0.64	0.72
ECS	0.60	0.23	0.33	0.59
SE	0.72	0.69	0.70	0.76
PSSM	0.72	0.63	0.67	0.75
ECS+SE	0.62	0.54	0.58	0.63
PSSM+ECS	0.78	0.74	0.76	0.86
PSSM+SE	0.84	0.71	0.77	0.88
Phy+ECS+SE	0.67	0.75	0.71	0.77
PSSM+ECS+SE	0.87	0.78	0.82	0.93
Phy+PSSM+ECS+SE	0.88	0.87	0.88	0.96
ASA+BC	0.63	0.61	0.62	0.69
Phy+ASA	0.65	0.73	0.69	0.75
Phy+BC	0.67	0.62	0.64	0.73
Phy+ASA+BC	0.68	0.67	0.68	0.77
Phy+ASA+BC+PSSM+ECS+SE	0.83	0.85	0.84	0.91

ASA denotes accessible surface area; BC denotes biochemical contacts; Phy means physicochemical features; ECS denotes evolutionary conservation score; SE means sequence entropy; and PSSM is the abbreviation of Position-Specific Scoring Matrix.

**Table 3:** The results of different models on 10-fold cross-validation test and independent test

Features	Testing	P	R	F1	AUC
ASA	10-fold	0.58	0.65	0.61	0.62
	Test set	0.60	0.61	0.61	0.57
BC	10-fold	0.66	0.33	0.43	0.67
	Test set	0.71	0.40	0.51	0.58
Phy	10-fold	0.63	0.51	0.55	0.67
	Test set	0.59	0.53	0.56	0.58
ECS	10-fold	0.58	0.27	0.34	0.51
	Test set	0.74	0.18	0.28	0.68
SE	10-fold	0.57	0.53	0.54	0.60
	Test set	0.59	0.61	0.60	0.52
PSSM	10-fold	0.65	0.54	0.58	0.65
	Test set	0.64	0.48	0.55	0.66
Phy+PSSM+ECS+SE	10-fold	0.65	0.65	0.65	0.68
	Test set	0.69	0.68	0.68	0.68
Phy+ASA+BC	10-fold	0.65	0.60	0.61	0.70
	Test set	0.62	0.70	0.66	0.62
Phy+ASA+BC+PSSM+ECS+SE	10-fold	0.66	0.68	0.66	0.72
	Test set	0.65	0.63	0.64	0.66

ASA denotes accessible surface area; BC denotes biochemical contacts; Phy means physicochemical features; ECS denotes evolutionary conservation score; SE means sequence entropy; and PSSM is the abbreviation of Position-Specific Scoring Matrix.

It can be seen that evolutionary conservation score plays a less important role in predicting hot spots. When compared with other features or feature combinations, the results obtained only from this feature are the least successful, with F1 = 0.33 and AUC = 0.59 with self-consistency test on training set. And on 10-fold cross-validation test, the F1 is 0.34 and the AUC is 0.51. However, when evolutionary conservation score is combined with physicochemical features, PSSM, and sequence entropy, there is at least 55% increase in F1 and 37% increase in AUC on self-consistency test. Similarly, the F1 increases from 0.34 to 0.65 and the AUC increases from 0.51 to 0.68 on 10-fold cross-validation test. Among sequence-based feature combinations, this feature combination mentioned above outperforms other sequence features combinations. The accessible surface area (ASA) feature performs better in predicting hot spots both on self-consistency test (F1 = 0.68 and AUC = 0.72) and 10-fold cross-validation test (F1 = 0.61 and AUC = 0.62). This suggests that a hot spot residue must be protected from bulk solvent (low relASA) [14], and might have largely relative change in ASA [50]. To our enjoyment, the combination of ASA, biochemical contacts, and physicochemical features performs better than feature ASA alone. The AUC has 5% and 8% increase on self-consistency test and 10-fold cross-validation test, respectively. The other feature related to structure is biochemical contacts. This term comprises hydrogen bonds, salt bridges, and atom contacts. Hot spots have more biochemical contacts than non-hot spots, because they contribute significantly to the binding free energy of complexes. The combination of ASA, biochemical contacts, and physicochemical features obtains the best performance among structure-based feature combinations. The F1 and AUC of this combination are 0.68 and 0.77 on self-consistency test, respectively. On 10-fold cross-validation test, this combination also obtains better results (F1 = 0.61 and AUC = 0.70). In conclusion, the sequence-based model obtains better performance both on self-consistency test (F1 = 0.88 and AUC = 0.96) and 10-fold cross-validation test (F1 = 0.65 and AUC = 0.68); the structure-based model is inferior both on self-consistency test (F1 = 0.68 and AUC = 0.77) and 10-fold cross-validation test (F1 = 0.61 and AUC = 0.70). The combination of all six features also obtains a better performance with F1 = 0.84 and AUC = 0.91 on self-consistency test. And on 10-fold cross-validation test, all features model achieves the best performance (F1 = 0.66 and AUC = 0.72). The analyses above indicate that, there is no single feature that makes a dominant contribution. Rather, it seems that the features in different combinations are complementary, and that the exploration of these complementarities might be very helpful for probing hot spots. It also supports the claim that there are no general patterns of hydrophobicity, shape or charge that can be used to easily detect hot spots [15,17].

We have also computed the contribution ratio of each feature in sequence-based model on 10-fold cross-validation test. Deleting one feature at a time can lead to some decrease in F1 score (see Table 4). Among excluded features, the physicochemical feature can make more decrease in F1 (ΔF = 0.06). The evolutionary conservation score shows a less contribution ratio in sequence-based model (ΔF = 0.01).

**Table 4:** Contribution ratio of each feature in sequence-based model on 10-fold cross-validation test

Excluded feature	P	R	F1	Δ**F1**
SE	0.63	0.62	0.62	-0.03
ECS	0.62	0.67	0.64	-0.01
PSSM	0.62	0.64	0.62	-0.03
Phy	0.61	0.58	0.59	-0.06

SE means sequence entropy; ECS denotes evolutionary conservation score; PSSM is the abbreviation of Position-Specific Scoring Matrix; and Phy means physicochemical features.

### Comparison with other methods on independent test set

To further validate the effectiveness of this sequence-based SVM approach, we compare our predictions with the predictions of other machine learning approaches on the same test set. This independent test set is obtained from Binding Interface Database (BID) [56], and contains 80 hot spots and 68 non-hot spots. These machine learning approaches are implemented on Weka platform [88]. We report the detailed results in Table 5. As observed from comparisons among these machine learning methods, the best prediction, in terms of F1 score, is achieved with RBFNNetwork method (F1 = 0.66). Nevertheless, these machine learning methods do not outperform SVMs approach (F1 = 0.68). The reason behind the differences of predictive performance possibly lies in the unbalanced distribution of two classes and the inapplicability of data set to some methods.

**Table 5:** Prediction results of machine-learning methods on independent test set

Method	P	R	F1
BaysNet	0.65	0.54	0.59
Logistic	0.62	0.63	0.62
RBFNNetwork	0.63	0.69	0.66
Decision Tree	0.66	0.60	0.63
Random Forest	0.68	0.55	0.61
Rules NNge	0.67	0.56	0.61
Lazy Kstar	0.63	0.51	0.57
Random Tree	0.61	0.50	0.55
Sequence-based SVM	0.69	0.68	0.68

Comparing with previously related studies on hot spots prediction is difficult, probably because data sets, hot spots definition, and evaluation measures are different. Moreover, it's not fair to compare the predictive power of methods based only on the quoted results. Therefore, we compare our prediction results with two energy-based methods Robetta [41] and FOLDEF [45], which are available via the internet on their web servers. The Robetta method is designed to predict the actual value of ΔΔG on the basis of a free energy function. In the original study, predicted and experimental hot spots are defined as those residues with ΔΔ*G *≥1 kcal.mol^-1^. We adopt this definition of a hot spot when training our model. The FOLD-X energy function (FOLDEF) method is developed to estimate the importance of the interactions which contribute to the stability of proteins and protein complexes. This method utilizes a full atomic description of the structure of the proteins. Also, it is based on energy function that takes into account different energy terms and predicts the change in interaction energy [45]. In FOLDEF computation results, a threshold of 1 kcal.mol^-1 ^is used to define predicted hot spots. The predicted results on independent test set are listed in Table 6. The F1 score is an effective metric to balance precision and recall rates, and gauges the relationship between them. The predictive performance of Robetta on independent test set is as follows: P = 0.66, R = 0.49 and F1 = 0.56. The FOLDEF method achieves an inferior result, with P = 0.50, R = 0.44 and F1 = 0.47. The sequence-based SVM method has the best results (F1 = 0.68). Cho's method gets the F1 of 0.57 on independent test set for the hot spots definitions of ΔΔ*G *≥1 kcal.mol^-1^.

**Table 6:** Comparison of different methods for hot spots prediction on independent test set

Method	P	R	F1
Robetta	0.66	0.49	0.56
FOLDEF	0.50	0.44	0.47
Cho's method	0.53	0.62	0.57
Sequence-based SVM	0.69	0.68	0.68
Structure-based SVM	0.62	0.70	0.66
All features SVM	0.65	0.63	0.64

### Prediction examples

**Figure 8:**
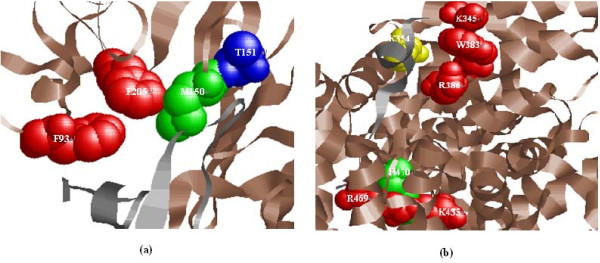
**Examples of hot spot prediction**. **(a) **Erythropoietin receptor/erythropoietin mimetic peptide (PDB ID: 1EBP, chain A). Red residues are actual hot spots predicted correctly. Blue residues are actual non-hot spots predicted correctly. Green residue indicates the residue that is an actual hot spot predicted as non-hot spot. Rasmol is used to graphically visualize the protein complexes in this study. **(b) **Adenomatous polyposis coli tumor suppressor protein/beta-catenin (PDB: 1JPP, chain B). Red residues are actual hot spots predicted correctly; green residue indicates the residue is an actual hot spot predicted as non-hot spot. Yellow residue is actual non-hot spot predicted as hot spot.

The X-ray crystal structure of a complex of EMP1 with the extracellular domain of the erythropoietin (EPO) receptor (EPO binding protein, EBP) (PDB: 1EBP) was previously reported by Livnah et al [89]. EMP1 is a peptide that is one of a series of related peptides discovered by phage display methodology, and possesses effective erythropoietin (EPO) mimetic action. Erythropoietin (EPO) is a hormone which regulates the cell proliferation and differentiation. EPO competes with EMP1 for receptor binding. Four residues in EPO receptor were analyzed through alanine scanning mutagenesis, and three out of four were found to be important for binding [90]. Experimentally identified hot spot residues in EPO receptor are F93, M150 and F205. T151 is experimentally assayed as a non-hot spot residue (from BID). The sequence-based SVM approach predicts two of three hot spots correctly (F93 and F205) and one non-hot spot (T151, see Figure 8a). While only one hot spot is incorrectly predicted as non-hot spot (M150). This corresponds to a better result with P = 1, R = 0.67, and F1 = 0.8. However, Robetta and FOLDEF methods predict M150 as hot spot correctly and other residues as non-hot spots.

A second example is the protein complex formed by beta-catenin and adenomatous polyposis coli (APC) (PDB: 1JPP) [91]. Beta-catenin is a cytosolic protein which has essential roles in cell adhesion and in the Wnt signaling pathway. The adenomatous polyposis coli (APC) tumor suppressor protein plays a critical role in regulating cellular levels of the oncogene product beta-catenin [92]. Beta-catenin has six experimentally identified hot spots (K345, W383, R386, K435, R469, and H470) and one non-hot spot (K354). Among seven residues, K345, K354, W383, and R386 form a cluster; K435, R469, and H470 form another cluster. Five of the six residues as hot spots (K345, W383, R386, K435, and R469) are predicted correctly, and one hot spot is incorrectly predicted as non-hot spot (H470). Whereas, one non-hot spot residue are incorrectly predicted as a hot spot (K354) (see Figure 8b). Our approach achieves a result with P = 0.83, R = 0.83, and F1 = 0.83. Robetta identifies all seven residues as non-hot spots. FOLDEF predicts K435 as a hot spot correctly and the rest as non-hot spots.

## Discussion

Many studies on the same problem of hot spots prediction have been published in the past years [20,40,48,50,64]. In these publications, hot spots are defined as those alanine mutations for which ΔΔ*G *≥2 kcal.mol^-1^. With this definition, our original training set comprises 84 hot spots and 293 non-hot spots. As mentioned above, comparing different approaches based on the quoted results is problematic as the definition of hot spots, data sets and evaluation measures used differ. It is not entirely fair to assess which methods perform better. Taking the Robetta for example, this method achieves F1 = 0.47 on our new training set (a threshold of ΔΔ*G *≥ 2 kcal.mol^-1^). The reported F1 score of Robetta in [20,48,50] and [64] on their respective training set are 0.49, 0.49, 0.55 and 0.59, respectively. This suggests that there will be different results with the same method on different training sets. Because of many differences, these methods can not be compared directly on the basis of the obtained results alone. Nonetheless, for completeness we compare our approach with two recent publications [50,64], Robetta and FOLDEF. The results are reported in Table 7. On the 2 kcal.mol^-1 ^training set, for the balance of data set we train our sequence-based method on the training set containing 84 hot spots and 84 selected non-hot spot with a lower ΔΔ*G*. The predictive performance of our method has been estimated to be P = 0.78, R = 0.80 and F1 = 0.79 on 10-fold cross-validation test. On training set, the sequence-based SVM achieves the best results (F1 = 0.79). While on independent test set, Tuncbag's method [64] obtains a better performance with F1 = 0.65. The results for sequence-based method appear comparable to those reported in Tuncbag's paper. While Cho's method performs better than two energy-based methods both on training set and independent test set.

**Table 7:** Predictive results of different methods when ΔΔ*G *≥ 2 kcal.mol^-1 ^is defined as a threshold

Method	Dataset	P	R	F1
Robetta	Training set	0.49	0.44	0.47
	Test set	0.71	0.25	0.37
FOLDEF	Training set	0.45	0.54	0.49
	Test set	0.60	0.26	0.37
Cho's method	Training set	0.58	0.73	0.65
	Test set	0.44	0.65	0.52
Tuncbag's method	Training set	0.64	0.52	0.57
	Test set	0.73	0.59	0.65
Sequence-based SVM	Training set	0.78	0.80	0.79
	Test set	0.65	0.64	0.64

Through comparisons of the results for different models, the sequence-based method has been found that its predictive performance is comparable to that of all features used method. However, the sequence-based model comprises only physicochemical features, position specific scoring matrix (PSSM), evolutionary conservation score, and sequence entropy without structure information. These features may be complementary, contain more hot spots' information, and depict the nature of hot spot residues. Thus, they can predict hot spots with better predictive performance. In a recent study [93], Westhead et al have shown that simple sequence-based features contain insufficient information and do not predict protein-protein interactions. However, these sequence features used in our work are not simple amino acid sequence, and they are derived from sequences.

We note that the evolutionary conservation score may not be a better predictor in distinguishing hot spots from non-hot spots by itself. In Ab+ dataset (all complexes), the statistically difference in conservation score between hot spots and non-hot spots is insignificant (p-value = 0.14). However, the difference in conservation score between hot spots and non-hot spots is statistically significant (p-value = 10^-4^) in Ab- dataset (excluding antigen-antibody complexes). When combined with other features, they can predict hot spots with better performance. As mentioned above, these features may be complementary and provide more amount of information. The predictive performance of the model based on evolutionary conservation score feature has been analyzed in Ab+ dataset (F1 = 0.34). Additionally, we have analyzed the predictive performance of the conservation score based method in Ab- dataset (F1 = 0.45, the data isn't listed). This suggests that evolutionary conservation score is not a good predictor when considering antigen-antibody complexes. As we all know, antibodies are easily mutated and diversified to neutralize various antigens.

We have analyzed the propensities of hot spots in antibody-antigen complexes and antibody proteins. In antibody-antigen complexes, hot spots are abundant in W, Y, and Q. While in antibody proteins, hot spots are enriched in I, N, and Q. Because there is a small number of antibody-antigen complexes in alanine scanning databank. These analyses are only based on our collecting data set. With the increase of alanine scanning data for antibody-antigen complexes, we may predict the hot spots in antibody-antigen complexes in the future.

In order to compare different models on a baseline, we have randomly selected 182 residues as hot spots from 377 interface residues and the rest are labeled as non-hot spots. With 10-fold cross-validation test, we have performed 10 times in whole experiment, and computed the equal value of each evaluation measure. The results are listed in Table 8. The F1 of sequence-based SVM is higher than that of random model (Δ F1 = 0.17) on 10-fold cross-validation test, and the difference is statistically significant (p-value = 0.003). Similarly, the F1 of structure-based SVM and all features SVM are also higher than that of random model (Δ F1 = 0.13 and 0.18, p-value = 0.02 and 0.002). These results indicate that three models obtain better predictive performance compared with a random model. Among these three models, all features-based SVM achieves the best results. However, the sequence-based SVM also gets comparable results without structure information.

**Table 8:** Comparisons of different models for hot spots prediction on 10-fold cross-validation test

Method	P	R	F1	Δ**F1**	P-value
Random	0.56	0.47	0.48	--	--
Sequence-based SVM	0.65	0.65	0.65	+0.17	0.003
Structure-based SVM	0.65	0.60	0.61	+0.13	0.02
All features SVM	0.66	0.68	0.66	+0.18	0.002

## Conclusions

In this work, we have presented a computational method, sequence-based SVM which combines strengths of machine learning and sequence information, to identify hot spots in protein-protein interfaces. The properties characterizing hot spot residues are various, and are not completely utilized by any one model [48]. Firstly, we analyzed the distributions of some features between hot spots and non-hot spots. We found that hot spots have lower relASA and larger relative change in ASA, suggesting hot spots tend to be protected from bulk solvent. With respect to biochemical contacts, hot spots have more contacts including hydrogen bonds, salt bridges, and atomic contacts, which favoring complexes formation. Not only conservation score but also sequence entropy doesn't differ significantly between hot spots and non-hot spots in Ab+ dataset. When antigen-antibody complexes are removed, there are significant differences in two features between hot pots and non-hot spots (p-value = 10^-4 ^for conservation score and p-value = 1.1 × 10^-3 ^for sequence entropy).

The combinations of different features have been explored as input vectors of machine learning method such as SVMs. We have noted that the sequence-based SVM approach exceeds other combinations of different features, with P = 0.69, R = 0.68, F1 = 0.68, and AUC = 0.68 on independent test set. Compared with several machine learning approaches, the sequence-based SVM approach is superior for identifying hot spots with reasonably predictive performance. In addition, the presented method is shown to exceed the prediction powers of two energy-based hot spots prediction approaches, for example, the Robetta and FOLDEF methods. Finally, we report two prediction examples: EMP1/EPO receptor complex and beta-catenin/adenomatous polyposis coli complex. The results indicate that our approach outperforms two energy-based approaches with high performance.

The sequence-based SVM method we have outlined here will assist in exploring protein interfaces and is a valuable tool capable of selecting target residues for alanine mutation, which is a complement to experimental investigation. This approach can predict hot spots without prior structural knowledge of the complex. Encouragingly, two previous studies have been reported in predicting hot spots based on sequence information [29,52]. Future progress depends as much on the application of novel computational approaches for analyzing protein interfaces and expanding the databank of alanine mutation [17]. Although systematic mutagenesis is currently expensive and time-consuming to perform, it is conceivable that recent developments in these aspects may greatly accelerate the process. On other hand, hot spots cannot be explained through simple analysis based on physicochemical characteristics, but there is reason to believe that more thorough computational approaches will succeed in capturing their essence [17]. For the future work, more efficient features will be explored and many machine learning methods will be used. The integration of features and machine learning methods will provide important insights in the field of drug discovery.

## Authors' contributions

RC participated in its design, performed the calculations, analyzed the data and drafted the manuscript. YT devised the concept and directed the research. WC, SY, and DW wrote the code and performed the calculations. YW, YT, and YS helped to draft the manuscript and finalized the draft. All authors read and approved the final manuscript.

## Supplementary Material

Additional file 1 **Training set. Table S1. Training set used in our work**. The measured ΔΔ G and the prediction from Robetta and FOLDEF are listed. The prediction results of our method are also reported. Click here for file

Additional file 2 **An independent test set. Table S2. Test set extracted from BID**. The effects of a mutation and the prediction from Robetta and FOLDEF are reported. Our predictions from sequence-based SVM model are also shown. Click here for file
